# Streptozotocin-Induced Cytotoxicity, Oxidative Stress and Mitochondrial Dysfunction in Human Hepatoma HepG2 Cells

**DOI:** 10.3390/ijms13055751

**Published:** 2012-05-11

**Authors:** Haider Raza, Annie John

**Affiliations:** Department of Biochemistry, Faculty of Medicine and Health Sciences, UAE University, POBox 17666, Al Ain, United Arab Emirates; E-Mail: anniej@uaeu.ac.ae

**Keywords:** streptozotocin, HepG2 cells, mitochondria, oxidative stress, GSH, ROS

## Abstract

Streptozotocin (STZ) is an antibiotic often used in the treatment of different types of cancers. It is also highly cytotoxic to the pancreatic beta-cells and therefore is commonly used to induce experimental type 1 diabetes in rodents. Resistance towards STZ-induced cytotoxicity in cancer cells has also been reported. Our previous studies have reported organ-specific toxicity and metabolic alterations in STZ-induced diabetic rats. STZ induces oxidative stress and metabolic complications. The precise molecular mechanism of STZ-induced toxicity in different tissues and carcinomas is, however, unclear. We have, therefore, investigated the mechanism of cytotoxicity of STZ in HepG2 hepatoma cells in culture. Cells were treated with different doses of STZ for various time intervals and the cytotoxicity was studied by observing the alterations in oxidative stress, mitochondrial redox and metabolic functions. STZ induced ROS and RNS formation and oxidative stress as measured by an increase in the lipid peroxidation as well as alterations in the GSH-dependent antioxidant metabolism. The mitochondria appear to be a highly sensitive target for STZ toxicity. The mitochondrial membrane potential and enzyme activities were altered in STZ treated cells resulting in the inhibition of ATP synthesis. ROS-sensitive mitochondrial aconitase activity was markedly inhibited suggesting increased oxidative stress in STZ-induced mitochondrial toxicity. These results suggest that STZ-induced cytotoxicity in HepG2 cells is mediated, at least in part, by the increase in ROS/RNS production, oxidative stress and mitochondrial dysfunction. Our study may be significant for better understanding the mechanisms of STZ action in chemotherapy and drug induced toxicity.

## 1. Introduction

Streptozotocin (STZ) is an antibiotic often used in the treatment of different types of cancers [1,2]. Structurally, it is an *N*-nitrosourea derivative of d-glucosamine that was first isolated from *Streptomyces achromogenes* [3]. It is a broad spectrum antibiotic and alkylating genotoxic agent which possesses antibacterial, tumoricidal, carcinogenic and diabetogenic properties [4–6]. However, STZ is not a drug of choice for treatment of cancers due to development of resistance to its genotoxic effects [7]. Moreover, severe toxicities were observed in different cancer patients when STZ was used alone or in combination with other antineoplastic drugs [8]. More specifically, STZ exhibits pancreatic beta-cell toxicity and is often used to induce diabetes in experimental animals [9–10]. Beta-cell toxicity and diabetogenic properties of STZ are mediated through diverse mechanisms including targeted uptake of STZ in beta cells by Glut2 receptors [11] and increased oxidative stress due to NO release and ROS production [12–16]. The exact mechanism of targeted beta-cell toxicity is, however, not clear. STZ induces beta-cell dysfunction and apoptosis at lower doses while causing beta-cell necrosis at higher doses. Insulin-secretory cells also develop resistance on repeated exposures to STZ through a wide spectrum of toxin tolerance mechanisms [17]. Betacyte, a genetically engineered insulin-secreting liver cell line derived from HepG2 cancer cells, known to exhibit general resistance to STZ, offers hope to elucidate the mechanism of action of beta-cell toxins in autoimmune destruction of the pancreas and in reversal of type 1 diabetes [18]. This study has also reported that the metabolic and morphological changes due to STZ treatment were similar in betacyte and HepG2 cells. In the present study, we have investigated dose- and time-dependent effects of STZ on HepG2 cell survival, mitochondrial function and oxidative stress related metabolic alterations. Our results have demonstrated that STZ induces cytotoxicity in HepG2 cells through increased oxidative stress and mitochondrial respiratory dysfunction with a limited induction of intrinsic mitochondrial apoptosis pathways.

## 2. Results and Discussion

### 2.1. Effect of STZ on HepG2 Cell Viability

**Figure 1:**
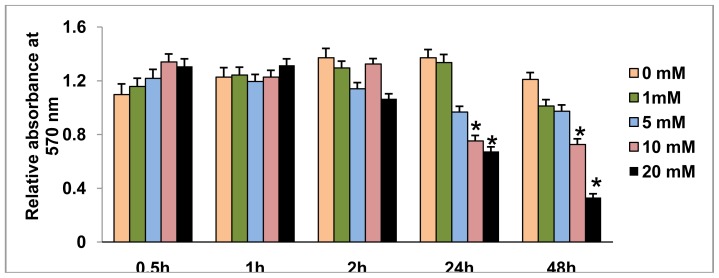
Cell viability assay by MTT. HepG2 cells (~5 × 10^4^) were grown in 96-well plates for 24 h and treated with different concentrations (0–20 mM) of STZ for different time intervals. The formazan crystals formed, following the reduction of MTT by metabolically active (viable) cells, were solubilized in acidified isopropanol and quantitated using the ELISA reader at 570 nm. Values are mean ± S.E.M. for three individual experiments. Asterisks indicate significant difference (*P* ≤ 0.05) relative to the untreated control cells.

A decrease in mitochondrial dehydrogenase based cell survival was observed only with higher concentrations of STZ (Figure 1). At low concentrations (1–5 mM) of STZ, no significant alteration in cell viability (5–10% changes) was observed over 24 h. Ten millimole per liter STZ treatment for 24 h or 48 h caused about 40% inhibition in cell viability. The maximum inhibition (~80%) was, however, observed in cells treated with 20 mM STZ for 48 h.

### 2.2. Effect of STZ on ROS, NO Production and LPO

**Figure 2:**
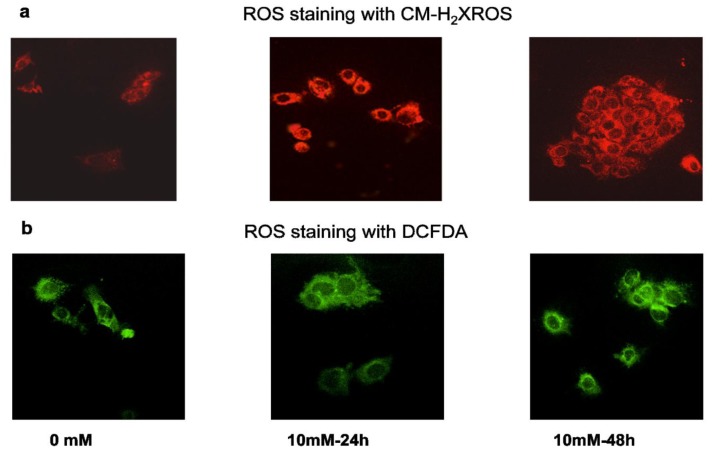
STZ-induced ROS production. HepG2 cells (1 × 10^5^) seeded in cover slip loaded 6-well plates were treated with medium containing 0.5 μM freshly prepared CM-H_2_XROS and incubated for 15 min at room temperature. After washing twice with PBS (pH 7.4) cells were fixed with 3.7% formaldehyde and visualized using an Olympus fluorescence microscope (**a**); Typical representations from untreated control and 10 mM STZ treated slides, from three individual experiments are shown. Intracellular production of reactive oxygen species was also measured in control untreated and STZ treated HepG2 cells using the cell permeable probe, DCFDA. Cells (~1 × 10^5^ cells/mL) were grown on cover slips and incubated with 5 μM DCFDA for 30 min at 37 °C. Cells were washed twice with PBS, and fluorescence was immediately analyzed microscopically as described above. Typical results from untreated control and 10mM STZ treated cells from three experiments are shown (**b**).

STZ causes a dose- and time-dependent increase in ROS production in HepG2 cells, which were observed microscopically and measured fluorimetrically (Figure 2). Increased ROS production in live HepG2 cells were captured microscopically using two different probes, CM-H_2_XROS and DCFDA, which again showed maximum fluorescence with 10 mM STZ at 48 h.

**Figure 3:**
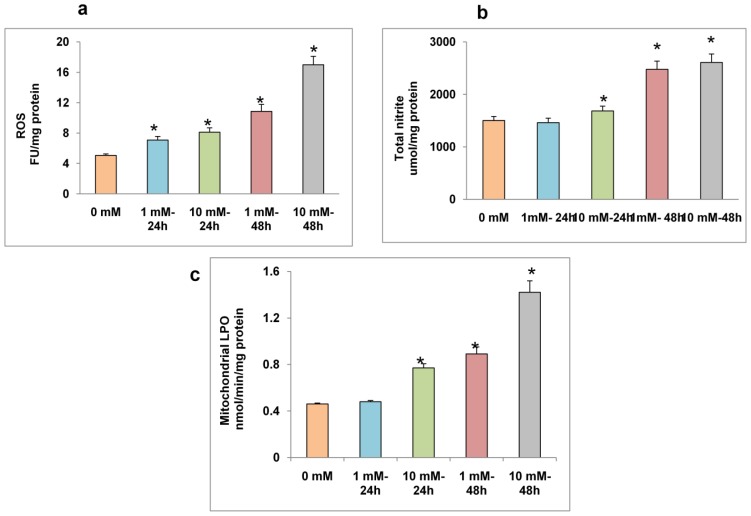
STZ-induced ROS, NO and LPO. Intracellular production of reactive oxygen species was measured fluorimetrically in control untreated and STZ treated HepG2 cells using DCFDA (**a**); For NO assay, HepG2 cells (2 × 10^5^ cells/well) were cultured in 6-well plates for 24 h prior to STZ treatments. NO production was determined by measuring the concentration of total nitrite in the culture supernatants (**b**) with Griess reagent (R & D Systems Inc.); NADPH-dependent-membrane LPO in the mitochondria of STZ treated HepG2 cells was measured as thiobarbituric acid reactive substances (TBARS) using malonedialdehyde as a standard (**c**). Results are expressed as mean ± S.E.M. of three independent experiments. Asterisks indicate significant difference (*P* ≤ 0.05) from untreated cells.

A time-dependent increase in ROS production was also measured fluorimetrically as shown in Figure 3a. Significant increases in ROS production were observed, with a marked increase (4-fold) observed with 10 mM STZ at 48 h. Figure 3b shows a time- and dose-dependent increase in NO production in STZ treated HepG2 cells. NO production was not significantly increased with 1 mM or 10 mM STZ after 24 h. On the other hand, 48 h treatments resulted in a marked increase (~70%) in NO production. A dose- and time-dependent increase in NADPH-dependent membrane bound LPO was also observed in HepG2 cells after STZ treatment (Figure 3c). These results clearly suggest that increased oxidative stress in STZ treated HepG2 cells is due to increased ROS/NO production.

### 2.3. Effects of STZ on Mitochondrial GSH Concentration and GSH Metabolism

**Figure 4:**
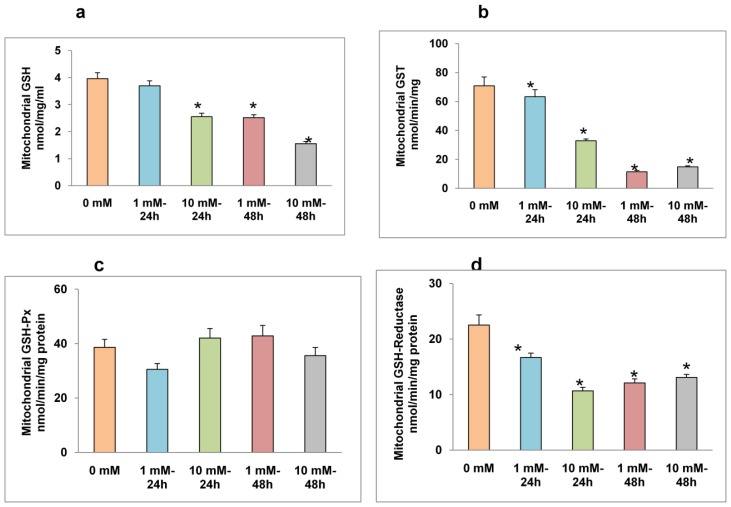
STZ-induced alterations in GSH metabolism. HepG2 cells were treated with different doses of STZ for different time intervals as given in the Materials and Methods. Mitochondrial GSH concentration (**a**); glutathione *S*-transferase (GST) (**b**); GSH-Px (**c**) and GSH-reductase (**d**) were measured. Results are expressed as mean ± S.E.M. of three independent experiments. Asterisks indicate significant difference (*P* ≤ 0.05) from untreated cells.

A dose-and time-dependent decrease in mitochondrial GSH was observed in HepG2 cells after STZ treatment (Figure 4a). More than a 2-fold decrease in mitochondrial GSH concentration was observed after 48 h of treatment with 10 mM STZ. Treatment with 1 mM STZ for 24 h showed little effect on the mitochondrial GSH pool. Similarly, a dose- and time-dependent inhibition of GSH conjugating enzyme, GST was observed after STZ treatment. 1 mM and 10 mM STZ treatments for 48 h resulted in a marked decrease (~4-fold) in GSH-conjugation of CDNB by GST enzymes (Figure 4b). As shown in Figure 4c, there was no significant alteration in mitochondrial GSH-Px activity after STZ treatment. On the other hand, a significant decrease in GSH-reductase activity was observed (Figure 4d). These results indicate that the reduced GSH pool in the mitochondria might be due to a decrease in regeneration of oxidized glutathione in the mitochondria or due to inhibition of cytosolic GSH transport to mitochondria in STZ treated HepG2 cells.

### 2.4. Effects of STZ on Mitochondrial Respiratory Function and ATP Production

**Figure 5:**
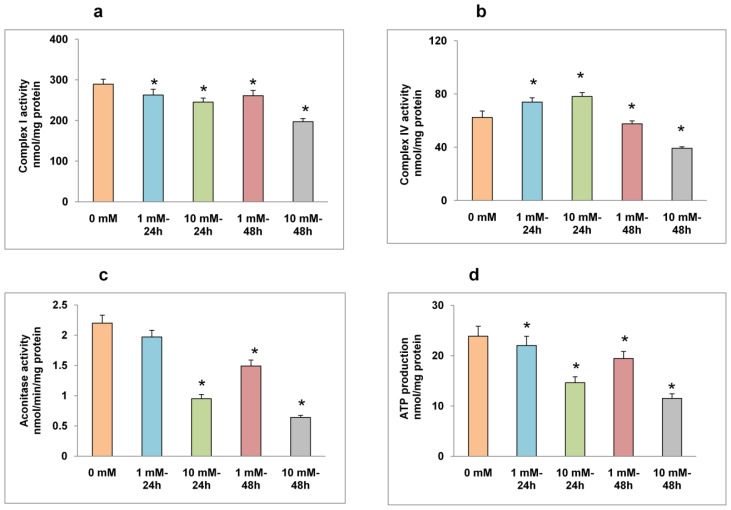
STZ-induced alterations in mitochondrial enzyme activity. Freshly isolated mitochondria from untreated control and STZ treated HepG2 cells were used to assay mitochondrial respiratory chain enzymes (**a**: Complex I, **b**: Complex IV) and the matrix enzyme (**c**: aconitase) activities as described in the Materials and Methods. ATP content (**d**) was measured in the total cell lysate using the ATP Bioluminescent somatic cell assay kit as described in the Materials and Methods. The values are expressed as mean ± S.E.M. of three independent experiments. Asterisks indicate significant difference (*P* ≤ 0.05) from untreated cells.

Alterations in mitochondrial respiratory enzymes and bioenergetics have also been observed in HepG2 cells after STZ treatment (Figure 5). Though significant inhibition in Complex I activity was observed after STZ treatment (Figure 5a), the activity of the terminal respiratory enzyme, Complex IV (cytochrome c oxidase) was markedly inhibited after 48 h of 10 mM STZ treatment (Figure 5b). A dose- and time-dependent inhibition of ROS-sensitive mitochondrial matrix enzyme, aconitase, was also observed after STZ treatment (Figure 5c). Similarly, ATP levels also decreased after STZ treatment (Figure 5d).

### 2.5. Effects of STZ on Cell Survival and Apoptosis

**Figure 6:**
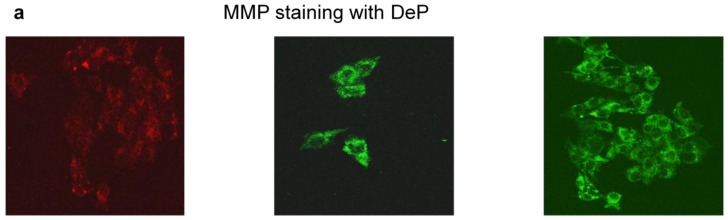

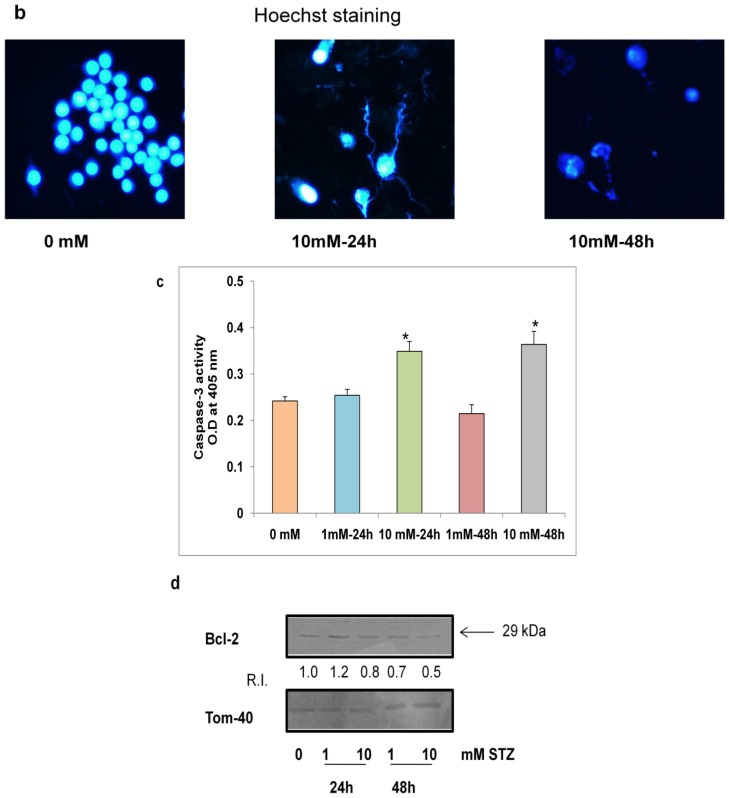
STZ-induced apoptosis and mitochondrial permeability transition. HepG2 cells were treated with different concentrations of STZ for different time intervals and membrane permeability transition (**a**) was measured using a cationic fluorescent dye (DePsipher™, R & D System Inc.) according to the vendor’s protocol. DePsipher has the property of aggregating upon membrane polarization forming an orange-red fluorescent (absorption/emission 585/590 nm) compound. If the membrane potential is reduced, the dye cannot access the transmembrane space and remains in its green fluorescent (510/527 nm) monomeric form. Results show a typical representation of three individual experiments, from untreated control and 10 mM STZ treated cells. Apoptosis measurement was performed by using Hoechst33342 dye staining of fragmented nuclei (**b**). Cover slips with adherent cells were treated with STZ, fixed with 3.7% formaldehyde and stained with Hoechst33342 (10 μg/mL) for 20 min at RT. The cover slips were washed, mounted on glass slides and analyzed by fluorescence microscopy. Cells with signs of apoptosis showed fragmented nuclei. Typical results from three experiments from untreated control and 10 mM STZ treated cells are presented. Caspase-3 activity in HepG2 cells treated with STZ was measured colorimetrically using the substrate DEVD peptide conjugated to p-nitroanaline as described in the vendor’s protocol (**c**). The values are expressed as mean ± S.E.M. of three independent experiments. Asterisks indicate significant difference (*P* < 0.05) from untreated cells. Mitochondrial extract (50 μg protein) from control untreated and STZ treated cells was separated on 12% SDS-PAGE and immunoblotted using Bcl-2 antibody (**d**). Tom-40 was used as a loading control. R.I. values indicate relative intensity of the protein band using expression of protein in control untreated cells as 1.0. The figure is representative of 2–3 experiments. Molecular weight (kDa) is indicated by an arrow.

STZ treatment resulted in induction of apoptosis in HepG2 cells as observed by a decrease in mitochondrial membrane potential (Figure 6a) and increased nuclear condensation (Figure 6b). An increase in apoptosis was also observed by an increased activity of terminal apoptotic enzyme caspase-3 (Figure 6c). This increased apoptosis after STZ treatment was apparently due to inhibition of expression of the antiapoptotic protein, Bcl-2 (Figure 6d).

### 2.6. Effect of STZ on the Expression of Apoptotic Protein Markers

**Figure 7:**
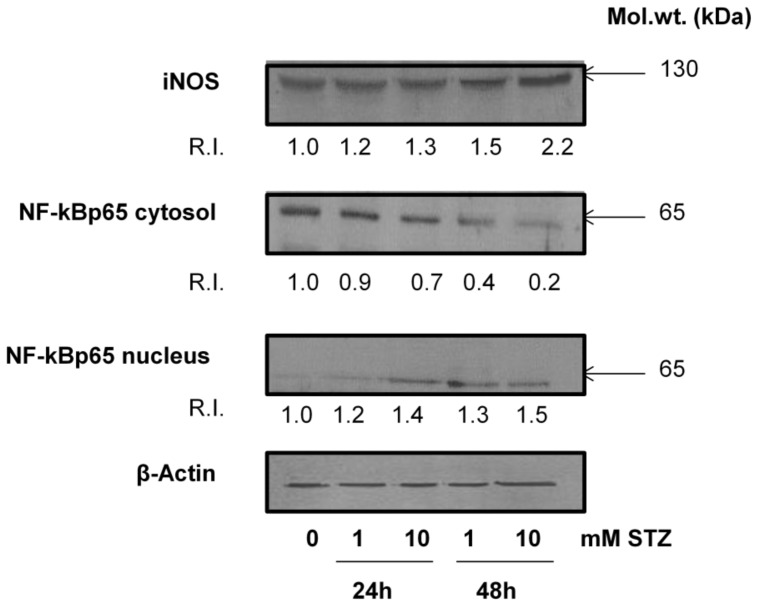
Expression of apoptotic markers. Cytosolic and nuclear extract (50 μg protein) from control untreated and STZ-treated HepG2 cells were separated on 12% SDS-PAGE and transferred on to nitrocellulose paper by Western blotting as described in the Materials and Methods. Specific antibodies against iNOS and NF-κBp65 were used to identify the expression of these proteins. Beta-actin was used as a loading control. R.I. values indicate relative intensity (of the protein band) using expression of proteins in control untreated cells as 1.0. The figures are representative of 2–3 experiments. Molecular weights (kDa) are indicated by arrows.

Figure 7 shows an increased expression of iNOS and increased translocation of NF-κBp65 from the cytosol to the nucleus after STZ treatment. These results are in agreement with the observation of increased oxidative and nitrosative stress after STZ treatment and that these changes might have altered the NF-κB-dependent cell signaling in HepG2 cells.

### 2.7. Discussion

STZ is a diabetogenic DNA alkylating agent that is commonly used in experimental models of type 1 diabetes in rodents [5,6]. Even therapeutic doses (up to 15 mM) of STZ induce pancreatic beta-cell death by inducing apoptosis followed by necrosis at higher doses (up to 30 mM) [19]. However, STZ treatment (up to 20 mM) has been reported to cause only apoptotic cell death in other cellular systems [20]. It has been reported that the increased STZ toxicity towards beta cells depends on the expression of GLUT2 receptors which preferentially facilitate the uptake of STZ [11]. Several *in vitro* studies using insulin secreting insulinoma cells, keratinocytes and genetically engineered hepatocytes have also shown that STZ (up to 20 mM) causes oxidative stress and apoptosis [11,18,20]. The precise molecular mechanism and metabolic targets of STZ toxicity in hepatocytes are not known. The mechanism of antineoplastic action of STZ in human hepatoma is also not clearly understood. We, therefore, have investigated the dose- and time-dependent effects of STZ on human hepatoma HepG2 cells, in culture. Using the mitochondrial dehydrogenase based cellular viability MTT assay, our results have shown that STZ induces significant cell death only after 48 h of treatment with 20 mM of the drug. Our results have also shown that 10 mM STZ resulted in about 40% cell death. At this dose, HepG2 cells exhibit increased ROS and NO production and an increase in LPO. The increase in oxidative stress is associated with increased apoptosis of HepG2 cells as evidenced by an increase in caspase-3 activity and reduction in the expression of antiapoptotic protein, Bcl-2, as also the nuclear fragmentation observed by Hoechst staining. A recent study has also suggested that the treatment of rat insulinoma cells (RINm5F) with STZ induces Bcl-2 dependent apoptosis which is protected by increasing the expression of this protein [21]. Several studies using *in vitro* cell culture or *in vivo* animal models, including our own, have suggested that STZ treatment causes an increase in oxidative stress and alterations in antioxidant GSH metabolism [12–15,22]. In the present study using HepG2 cells, we have observed a reduced mitochondrial GSH antioxidant pool due to the reduced recycling of oxidized glutathione resulting from the inhibition of mitochondrial GSH-reductase enzyme. We have also demonstrated that the increased ROS production and reduced GSH concentration in HepG2cells treated with STZ has resulted in compromised mitochondrial bioenergetics. Mitochondrial membrane potential was reduced after STZ treatment which resulted in inhibition of the activities of the respiratory complexes and ROS-sensitive aconitase enzyme followed by a reduction in ATP synthesis. Our *in vivo* studies have also shown increased mitochondrial dysfunction after STZ treatment [14,15]. In the present study, we have also provided evidence that the increased oxidative stress, apoptosis and mitochondrial dysfunction in HepG2 cells might be associated with altered NF-κB based cell signaling as STZ increases the expression of iNOS and translocation of NF-κBp65 (RelA) transcription factor to the nucleus. STZ has been reported to alter NO/NF-κB dependent cell signaling [23,24]. STZ methylates DNA both under *in vitro* and *in vivo* conditions and this methylated DNA may have altered regulation of DNA transcription in drug treated cells [25,26].

## 3. Experimental Section

### 3.1. Materials

Streptozotocin (STZ), cytochrome c, reduced and oxidized glutathione (GSH), 5,5′-dithio-*bis*(2-nitrobenzoic acid), 1-chloro 2,4-dinitrobenzene (CDNB), cumene hydroperoxide, glutathione reductase, thiobarbituric acid, 3-(4,5-dimethylthiazol-2-yl)-2,5-diphenyltetrazolium bromide (MTT), NADH, NADPH, coenzyme Q_2_, antimycin A, dodecyl maltoside, Hoechst 33342 and ATP bioluminescent somatic cell assay kits were purchased from Sigma-Aldrich Fine Chemicals (St Louis, MO, USA). 2′,7′-Dichlorofluorescein diacetate (DCFDA) and CM-H_2_XROS were procured from Molecular Probes (Eugene, OR, USA). Aconitase assay kit was purchased from Oxis International Inc. (Portland, OR, USA). Kits for mitochondrial membrane potential, nitric oxide and caspase-3 assays were purchased from R & D System, MN, USA. HepG2 cells were obtained from American Type Culture Collection (Manassas, VA, USA). Polyclonal antibodies against beta-actin, Tom-40, NF-κB, iNOS and Bcl-2 were purchased from Santa Cruz Biotechnology Inc. (Santa Cruz, CA, USA). Reagents for cell culture, SDS-PAGE and Western blot analyses were purchased from Gibco BRL (Grand Island, NY, USA) and Bio Rad Laboratories (Richmond, CA, USA).

### 3.2. HepG2 Cell Culture and STZ Treatment

HepG2 cells were grown in poly-l-lysine coated 75 cm^2^ flasks (~2.0–2.5 × 10^6^ cells/mL) in DMEM medium supplemented with 1% nonessential amino acids, 2 mM glutamine, 10% heat inactivated fetal bovine serum in a humidified incubator in the presence of 5% CO_2_–95% air at 37 °C. HepG2 cells were treated with 0–20 mM STZ (dissolved in citrate buffer, pH 4.4 and diluted in DMEM to appropriate concentrations just before use) for different time intervals (24 to 48 h). Control cells were treated with vehicle alone. Concentrations and time points for STZ treatment in this study were based on MTT cytotoxicity tests and on published reports on *in vitro* and *in vivo* use of STZ [18,19].After the desired time of treatment, cells were harvested, washed with PBS (pH 7.4) and homogenized in H-medium buffer (70 mM sucrose, 220 mM mannitol, 2.5 mM HEPES, 2 mM EDTA, and 0.1 mM phenylmethylsulfonylfluoride, pH 7.4) at 4 °C Cellular fractionation to prepare mitochondria and postmitochondrial (PMS) fractions were performed by centrifugation and the purity of the isolated fractions for cross contaminations was checked as described before [27].

### 3.3. MTT Cell Viability Test

The effect of different concentrations of STZ on cell viability test was evaluated based on the metabolic activity of mitochondrial dehydrogenases, which cleave the dye MTT to form purple formazan crystals as described previously [27].

### 3.4. Measurement of ROS, NO and LPO

To evaluate direct production of mitochondrial ROS in HepG2 cells, ROS-specific staining with Mito Tracker Red (CM-H_2_XROS) was used. Respiring cells which produce ROS (mainly H_2_O_2_) convert (oxidize) the dye to a cationic form, which interacts with mitochondrial proteins and generates red fluorescence. Briefly, HepG2 cells (1 × 10^5^) were seeded in cover slip loaded 6-well plates. After treatment with appropriate doses of STZ for appropriate time intervals, the cells were then treated with medium containing 0.5 μM freshly prepared CM-H_2_XROS and incubated for 15 min at room temperature. After washing twice with PBS (pH 7.4), cells were fixed with 3.7% formaldehyde and mounted on to microscope slides and visualized using the Olympus fluorescence microscope. A typical result showing red stained cells has been shown.

Intracellular production of reactive oxygen species was also measured using the cell permeable probe, DCFDA, which preferentially measures peroxides. Briefly, STZ treated and control cells (~1 × 10^5^ cells/mL) were grown on cover slips and incubated with 5 μM DCFDA for 30 min at 37 °C. Cells were washed twice with PBS, and fluorescence was immediately analyzed microscopically as described above. DCFDA based ROS assay was also performed and measured fluorimetrically as described before [15].

For NO assay, HepG2 cells (2 × 10^5^ cells/well) were cultured in 6-well plates for 24 h prior to STZ treatments. NO production was determined by measuring the concentration of total nitrite in the culture supernatants using Griess reagent (R & D Systems Inc.).

NADPH-dependent-membrane lipid peroxidation in the mitochondria of STZ treated and control HepG2 cells was measured as thiobarbituric acid reactive substances (TBARS) using malonedialdehyde as standard as described previously [27,28].

### 3.5. Measurement of GSH Metabolism

HepG2 cells were treated with different doses of STZ for appropriate time intervals as mentioned above. Mitochondrial GSH concentration and activities of GSH-Px, GSH-reductase and glutathione *S*-transferase (GST) were measured in the isolated mitochondria as described before [28].

### 3.6. Measurement of Mitochondrial Functions

#### 3.6.1. Measurement of Mitochondrial Membrane Potential (MMP)

Loss of mitochondrial membrane potential was measured in HepG2 cells after treatment with varying concentrations of STZ at different time intervals using a fluorescent cationic dye, DePsipher™ (R&D System Inc.) according to the vendor’s protocol. DePsipher has the property of aggregating upon membrane polarization forming an orange-red fluorescent (absorption/emission 585/590 nm) compound. If membrane potential is disturbed, the dye cannot access the transmembrane space and remains in its green fluorescent (510/527 nm) monomeric form. Briefly, cells were grown on coverslips as described above and adherent cells were treated with STZ and then incubated with DePsipher reagent in pre-warmed medium for 20 min in a 37 °C incubator. Cover slips were washed with PBS and fixed with fresh 3.7% formaldehyde. Healthy cells containing red aggregates were differentiated from apoptotic cells (containing only green monomers) by fluorescence microscopy. A typical result from three experiments is presented.

#### 3.6.2. Measurement of Mitochondrial Respiratory Functions and ATP Content

Freshly isolated mitochondria (5 μg protein) from untreated and STZ-treated HepG2 cells were suspended in 1.0 mL of 20 mM potassium phosphate buffer, pH 7.4, in the presence of the detergent, lauryl maltoside (0.2%). NADH-ubiquinone oxidoreductase (NADH-dehydrogenase (complex I) and cytochrome c oxidase (complex IV) were measured using the substrates CoenzymeQ_2_ and reduced cytochrome c, respectively, by the methods of Birch-Machin and Turnbull [29].

Mitochondrial aconitase activity was measured by the NADPH coupled conversion of citrate to isocitrate in the presence of isocitrate dehydrogenase using the Bioxytech Aconitase-340 assay kit or by using the method described by Spear *et al.* [30]. Aconitase activity was expressed as the rate of formation of NADPH determined at 340 nm.

The ATP content in the STZ-treated and untreated HepG2 cell lysates was determined using an ATP Bioluminescent cell assay kit according to the manufacturer’s recommended protocol and samples were analyzed using the TD-20/20 Luminometer (Turner Designs, Sunnyvale, CA). A standard curve with concentrations of ATP ranging from 0–200 nmol/mL was used for the assay [27].

### 3.7. Measurement of Apoptosis

#### 3.7.1. Nuclear Staining with Hoechst 33342

Apoptosis measurement was performed by Hoechst dye staining of fragmented nuclei. Cover slips with adherent cells were treated with STZ and cells were fixed with 3.7% formaldehyde & stained with Hoechst33342 (10 μg/mL) for 20 min at room temperature. The cover slips were washed, mounted on glass slides and analyzed by fluorescence microscopy. Cells with signs of apoptosis showed fragmented nuclei. A typical result from three experiments is presented.

#### 3.7.2. Assay of Caspase-3 Activity

Apoptosis was also measured by measuring the activity of caspase-3 enzyme. HepG2 cells (2 × 10^6^ cells/well), grown in 6-well plates, were treated with increasing concentrations of STZ as described above. Caspase-3 activity was measured in the cell lysate using the caspase-specific peptide substrate, DEVD, conjugated to reporter p-nitroanaline molecules. Cleavage of this peptide by caspase releases the chromophore which is measured colorimetrically at a wavelength of 405 nm as described in the vendor’s protocol (R & D Systems) as published before [27].

### 3.8. SDS-PAGE and Western Blot Analysis

Proteins (50 μg) were separated on 12% SDS-PAGE [31] and electrophoretically transferred on to nitrocellulose paper by Western blotting [32]. The immunoreactive protein bands were visualized after interacting with primary antibodies against iNOS, Bcl-2 and NF-κB p65. Immunoreactive bands were visualized using the appropriate conjugated secondary antibodies. Densitometric analysis of the protein bands was performed using a gel documentation system (Vilber Lourmat, France) and expressed as relative intensity (R.I.) compared to the untreated control.

### 3.9. Statistical Analysis

Values shown are expressed as mean ± S.E.M. of three individual experiments. Statistical significance of the data was assessed using SPSS software by analysis of variance followed by Dunnett analysis. *P* values ≤ 0.05 were considered statistically significant.

## 4. Conclusions

In summary, our results have shown that STZ induces apoptosis in HepG2 cancer cells which is associated with increased oxidative and nitrosative stress and mitochondrial dysfunction. The study also provides evidence that GSH-dependent antioxidant metabolism is adversely affected, causing imbalance in NF-κB dependent redox signaling and mitochondrial bioenergetics. These results may be significant for understanding the role of STZ as an antineoplastic and/or diabetogenic agent and its associated toxicities in different tissues.

## Abbreviations

DCFDA: 2′,7′-dichlorofluorescein diacetate
GSH: glutathione
GST: glutathione *S*-transferase
GSH-Px: glutathione peroxidase
LPO: lipid peroxidation
MMP: mitochondrial membrane potential
NO: nitric oxide
ROS: reactive oxygen species
STZ: streptozotocin
SDS-PAGE: sodium dodecylsulphate polyacrylamide gel electrophoresis
